# Endogenous GLP-1 mediates postprandial reductions in activation in central reward and satiety areas in patients with type 2 diabetes

**DOI:** 10.1007/s00125-015-3754-x

**Published:** 2015-09-18

**Authors:** Jennifer S. ten Kulve, Dick J. Veltman, Liselotte van Bloemendaal, Frederik Barkhof, Carolyn F. Deacon, Jens J. Holst, Robert J. Konrad, John H. Sloan, Madeleine L. Drent, Michaela Diamant, Richard G. IJzerman

**Affiliations:** Department of Internal Medicine, Diabetes Center, VU University Medical Center, de Boelelaan 1117, 1081 HV Amsterdam, the Netherlands; Department of Psychiatry, VU University Medical Center, Amsterdam, the Netherlands; Department of Radiology and Nuclear Medicine, VU University Medical Center, Amsterdam, the Netherlands; The NNF Center for Basic Metabolic Research, Department of Biomedical Sciences, Panum Institute, University of Copenhagen, Copenhagen, Denmark; Lilly Research Laboratories, Eli Lilly and Company, Indianapolis, IN USA; Department of Internal Medicine/Endocrine Section, VU University Medical Center, Amsterdam, the Netherlands; Department of Clinical Neuropsychology, VU University, Amsterdam, the Netherlands

**Keywords:** fMRI, Food intake, GLP-1, Neuroimaging, Obesity, Type 2 diabetes

## Abstract

**Aims/hypothesis:**

The central nervous system (CNS) is a major player in the regulation of food intake. The gut hormone glucagon-like peptide-1 (GLP-1) has been proposed to have an important role in this regulation by relaying information about nutritional status to the CNS. We hypothesised that endogenous GLP-1 has effects on CNS reward and satiety circuits.

**Methods:**

This was a randomised, crossover, placebo-controlled intervention study, performed in a university medical centre in the Netherlands. We included patients with type 2 diabetes and healthy lean control subjects. Individuals were eligible if they were 40–65 years. Inclusion criteria for the healthy lean individuals included a BMI <25 kg/m^2^ and normoglycaemia. Inclusion criteria for the patients with type 2 diabetes included BMI >26 kg/m^2^, HbA_1c_ levels between 42 and 69 mmol/mol (6.0–8.5%) and treatment for diabetes with only oral glucose-lowering agents. We assessed CNS activation, defined as blood oxygen level dependent (BOLD) signal, in response to food pictures in obese patients with type 2 diabetes (*n* = 20) and healthy lean individuals (*n* = 20) using functional magnetic resonance imaging (fMRI). fMRI was performed in the fasted state and after meal intake on two occasions, once during infusion of the GLP-1 receptor antagonist exendin 9-39, which was administered to block actions of endogenous GLP-1, and on the other occasion during saline (placebo) infusion. Participants were blinded for the type of infusion. The order of infusion was determined by block randomisation. The primary outcome was the difference in BOLD signal, i.e. in CNS activation, in predefined regions in the CNS in response to viewing food pictures.

**Results:**

All patients were included in the analyses. Patients with type 2 diabetes showed increased CNS activation in CNS areas involved in the regulation of feeding (insula, amygdala and orbitofrontal cortex) in response to food pictures compared with lean individuals (*p* ≤ 0.04). Meal intake reduced activation in the insula in response to food pictures in both groups (*p* ≤ 0.05), but this was more pronounced in patients with type 2 diabetes. Blocking actions of endogenous GLP-1 significantly prevented meal-induced reductions in bilateral insula activation in response to food pictures in patients with type 2 diabetes (*p* ≤ 0.03).

**Conclusions/interpretation:**

Our findings support the hypothesis that endogenous GLP-1 is involved in postprandial satiating effects in the CNS of obese patients with type 2 diabetes.

*Trial registration*: ClinicalTrials.gov NCT 01363609

*Funding* The study was funded in part by a grant from Novo Nordisk.

## Introduction

The role of the central nervous system (CNS) in the regulation of energy balance involves a complex interaction of signals originating from the periphery (i.e. hormones and neuronal signals) and responses of brain areas involved in the reward and regulation of food intake [1]. An excess of food intake compared with energy expenditure induces a chronically positive energy balance causing weight gain and obesity. In the search for strategies to treat and prevent obesity, it is important to increase understanding of the central regulation of feeding and the physiological signals influencing this regulation.

Hormones derived from the gut appear to relay meal-related information on nutritional status to the CNS, thereby affecting feeding [2]. The gut hormone glucagon-like peptide-1 (GLP-1) is released by enteroendocrine L cells into the circulation following food ingestion. GLP-1 is known for its incretin effect, as it augments meal-related insulin secretion from the pancreas [3]. In addition, results from preclinical and clinical studies demonstrate that administration of GLP-1 or GLP-1 receptor agonists (GLP-1RA) in pharmacological amounts reduces appetite, food intake and body weight [4–8]. In animal studies, the pharmacological effects of GLP-1RA are at least partly mediated through the CNS [9–15]. Results from studies in rodents and humans demonstrate that endogenous GLP-1 (i.e. at lower levels of GLP-1 compared with pharmacological administration of GLP-1RA) plays a role in the regulation of food intake [16, 17]. Although studies in rodents indicate that this effect is also mediated via the CNS [11, 18], the involvement of endogenous GLP-1 in the central regulation of food intake in humans has not been investigated.

Neuroimaging techniques enable non-invasive investigation of the CNS in humans. Functional magnetic resonance imaging (fMRI) can be used to measure food-cue related changes in activity in the CNS. Obese individuals show increased activation when viewing food pictures [19] and in particular pictures of high-energy food [20, 21]. In addition, it was shown that food intake reduces CNS activation in response to viewing food pictures [22]. Furthermore, we recently demonstrated that acute administration of pharmacological amounts of a GLP-1RA diminishes activation to food pictures in areas involved in the regulation of food intake [19].

In the present study, we used fMRI to assess the physiological role of GLP-1 in the central regulation of food intake in obese patients with type 2 diabetes and healthy lean individuals. We measured CNS activation in response to viewing food pictures before and after intake of a meal on two test visits. During one of the visits, the GLP-1 receptor antagonist exendin 9-39 was administered to evaluate the effects of endogenous GLP-1. We hypothesised that the satiating effects of meal intake on CNS activation would be prevented by blocking endogenous GLP-1.

## Methods

### Participants

The study was approved by the Medical Ethics Review Committee of the VU University Medical Center (VUMC) and conducted in accordance with the Declaration of Helsinki. All participants provided written informed consent. The study included 20 overweight and obese patients with type 2 diabetes and 20 healthy lean individuals matched for sex and age. Individuals were eligible if they were 40–65 years of age and right-handed. Inclusion criteria for the healthy lean individuals included a BMI <25 kg/m^2^ and normoglycaemia, defined by fasting plasma glucose <5.6 mmol/l and 2 h glucose <7.8 mmol/l following a 75 g oral glucose tolerance test. Inclusion criteria for the patients with type 2 diabetes included BMI >26 kg/m^2^, HbA_1c_ levels between 42 and 69 mmol/mol (6.0–8.5%) and treatment for diabetes of the oral glucose-lowering agents metformin ± sulfonylurea. Exclusion criteria were a history of neurological, cardiovascular, renal or liver disease, malignancies, the use of any centrally acting agent, substance abuse and psychiatric disorders. All patients with diabetes were treated with metformin and 12 patients were also treated with sulfonylurea, but sulfonylurea were temporarily discontinued 4 weeks prior to the start of the experiments. Ten patients used antihypertensive medication and 15 patients used cholesterol-lowering agents.

### General experimental protocol

**Fig. 1:**
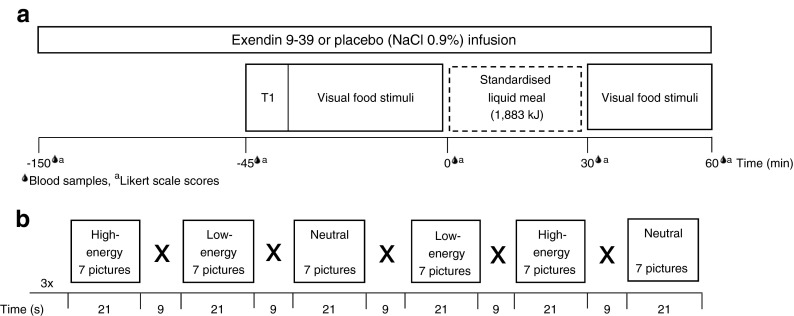
Study protocol. (**a**) Study design. Obese patients with type 2 diabetes and healthy lean individuals were studied in a placebo-controlled acute intervention study. The study consisted of two visits (random order): one with a GLP-1 receptor antagonist (exendin 9-39) infusion and one with a saline (placebo) infusion. Infusions started 1 h before the scan and lasted until the end of the visit. During each visit, two fMRI scans were performed: one while fasted and one 30 min after the meal intake. During fMRI, visual-food cues were presented. Blood samples and appetite-related scores on a 10-point Likert scale were taken at fixed time points. T1, structural MRI, T1-weighted sequence. (**b**) fMRI paradigm. One run comprised six blocks of 21 s each (seven pictures). Within one run, two blocks of each category were presented. Each MRI session included three runs

This was a placebo-controlled, crossover, acute intervention study. The study consisted of two separate test visits. On each visit, two fMRI scans were performed; one while the individual fasted and one at 30 min after intake of a standardised liquid meal consisting of 1,883 kJ (carbohydrate 56.1 g, fat 17.4 g and protein 18.0 g, 300 ml Nutridrink yoghurt style, Nutricia, Zoetermeer, the Netherlands). At each visit, a catheter was inserted into a cubital vein for infusion of, in random order, either placebo (sodium chloride 0.9% wt/vol.) or the selective GLP-1 receptor antagonist exendin 9-39 (600 pmol kg^−1^ min^−1^; Clinalfa, Bachem, Bubendorf, Switzerland, used to block the effects of endogenous GLP-1), using a MRI-compatible infusion pump (MRIdium 3850 MRI-IV pump, Iradimed, Winter Park, FL, USA). The order of infusion was determined by block randomisation. Each infusion was started 1 h before the beginning of the MRI scan and was continued during the whole period of scanning. The participants were blinded for the type of infusion. Blood was drawn at fixed intervals to measure glucose, GLP-1, insulin and glucagon levels (Fig. 1a).

### fMRI paradigm

The fMRI task has been described previously [19]. Briefly, the fMRI task consisted of pictures selected from three different categories: (1) high-energy food items; (2) low-energy food items; (3) non-food items. The pictures were presented via the software E-prime 1.2 (Psychology Software Tools, Pittsburgh, PA, USA). Forty-two pictures per category were presented in a block design (Fig. 1b). The order of the blocks was randomised. Given that each participant was scanned four times, four versions of this paradigm were created with different pictures, with the images being matched between the versions and between the categories for type, shape and colour.

### MRI acquisition and analyses

Comparable MRI acquisition and analyses were used as described previously [19]. In brief, MRI data were acquired on a 3.0 Tesla GE Signa HDxt scanner (General Electric, Milwaukee, WI, USA) and fMRI data were acquired using an echo planar imaging T2* blood oxygen level dependent (BOLD) pulse-sequence. Functional images were analysed with SPM8 software (Wellcome Trust Centre for Neuroimaging, London, UK). At the first level, high-energy food, low-energy food and non-food blocks were modelled. Next, we computed two contrasts of interest: all food pictures > non-food pictures; high-energy food pictures > non-food pictures. These first-level contrast images were entered into second-level three-way ANOVA with factors group (healthy lean, diabetes), infusion (placebo, exendin 9-39) and meal state (fasted, postprandial). A priori regions of interest (ROIs) were determined based on previous studies (i.e. left and right insula, caudate nucleus, putamen, amygdala and orbitofrontal cortex (OFC)) [19–21]. CNS activations are reported as significant when they survive family-wise error correction for multiple comparisons on the voxel level using small volume correction within predefined ROIs, as described previously [19].

### Blood sampling and assays

Measurement of blood glucose was performed using the glucose dehydrogenase method (GlucoseAnalyser, HemoCue, Ängelholm, Sweden). Total GLP-1 was analysed using a C-terminally directed radioimmunoassay for amidated GLP-1 (antibody 89390) [23]. Insulin levels were measured using an immunometric assay (Advia Centaur, Siemens Medical Solutions Diagnostics, Tarrytown, NY, USA). Glucagon levels were determined using an immunoassay as described previously (Lilly Research Laboratories, Indianapolis, IN, USA) [24].

### Questionnaires

The participants were asked to score their sensations of hunger, fullness, prospective food consumption and nausea on a 10-point Likert scale at four fixed time points during the visits: (1) before the start of the first (fasted) MRI session; (2) before intake of the meal; (3) 30 min after meal intake; (4) 60 min after meal intake. Changes in scores from before meal intake to 30 and 60 min after intake were analysed and compared between infusions.

### Statistical analyses

Clinical group data were analysed with the Statistical Package for the Social Sciences version 20 (IBM SPSS Statistics for Windows, Version 20.0. Armonk, NY, USA). Data are expressed as mean ± SEM (unless otherwise stated). Between-group differences were analysed with independent Student’s *t* test. In cases of measurements with more than one time point on each visit, repeated measures ANOVA was used with time (min) as the within-subject factor and group as the between-subject factor, or treatment as the within-subject factor. Results were considered statistically significant when *p* < 0.05.

## Results

### Baseline characteristics

Table 1 summarises the baseline characteristics of both groups. All participants completed all visits. Due to a technical failure, one postprandial scan of a patient with diabetes during the visit with exendin 9-39 infusion could not be used in the analysis.

**Table 1:** Baseline characteristics

Characteristic	Healthy controls (*n* = 20)	Obese T2DM patients (*n* = 20)	*p* value
Age (years)	56.3 ± 1.4	59.5 ± 0.9	0.06
Sex, male/female (*n*)	10/10	11/9	0.8
Weight (kg)	69.9 ± 2.5	95.4 ± 3.4	<0.001
BMI (kg/m^2^)	22.5 ± 0.4	32.0 ± 1.1	<0.001
Waist circumference (cm)	81.4 ± 1.8	108.9 ± 2.5	<0.001
Body fat (%)	24.7 ± 1.3	38.6 ± 1.8	<0.001
Systolic BP (mmHg)	113 ± 3.5	128 ± 2.0	0.001
Diastolic BP (mmHg)	72.9 ± 2.5	78 ± 1.8	0.1
HbA_1c_ (mmol/mol)	37 ± 0.4	56 ± 2.2	<0.001
HbA_1c_ (%)	5.5 ± 0.03	7.3 ± 0.2	<0.001
Fasting plasma glucose (mmol/l)	5.2 ± 0.1	8.4 ± 0.3	<0.001
Total cholesterol (mmol/l)	5.2 ± 0.2	4.5 ± 0.3	0.07
Triacylglycerol (mmol/l)	0.8 ± 0.1	1.6 ± 0.1	<0.001
Diabetes duration (years)	–	7.8 ± 1.1	–
BP-lowering medications (*n*)	0	10	<0.001
Cholesterol-lowering medications (*n*)	0	15	<0.001

Data are means ± SEM or number of individuals (*n*)

T2DM, type 2 diabetes

The effects of group, meal intake and GLP-1 receptor blockade on CNS activation are presented in Table 2.

**Table 2:** Effects of group, meal intake and GLP-1 receptor blockade on CNS activation in response to viewing of food pictures and high-energy food pictures

Contrast used	Comparison	Region	Side	Cluster	*Z*	FWE *p* value	MNI coordinates (x, y, z)
Group differences							
Food > non-food	Healthy controls > T2DM (fasted, placebo)	–	–	–	–	–	–
High-energy > non-food		–	–	–	–	–	–
Food > non-food	T2DM > Healthy controls (fasted, placebo)	Amygdala	L	13	2.79	0.02	−27, −4, −17
		Insula	R	41	3.24	0.02	29, 2, −14
		Insula	L	13	2.99	0.04	−30, 14, −17
		OFC	R	61	3.77	0.004	39, 26, −11
High-energy > non-food		Insula	L	19	3.21	0.02	−30, 14, −14
		OFC	R	16	2.87	0.05	42, 29, −11
Food > non-food	Healthy controls > T2DM (postprandial, placebo)	–	–	–	–	–	–
High-energy > non-food		–	–	–	–	–	–
Food > non-food	T2DM > Healthy controls (postprandial, placebo)	–	–	–	–	–	–
High-energy > non-food		–	–	–	–	–	–
Meal effects							
Food > non-food	Healthy controls: fasted > postprandial (placebo)	Insula	R	9	3.16	0.02	36, −16, 7
High-energy > non-food		Insula	R	6	2.67	0.08	36, −16, 7
Food > non-food	T2DM: fasted > postprandial (placebo)	Insula	R	11	3.00	0.04	39, 2, −14
		Insula	L	24	2.85	0.05	−42, 11, −8
High-energy > non-food		Caudate nucl.	L	38	3.58	0.007	−12, 23, 1
		Insula	L	23	2.96	0.04	−36, 8, −14
		OFC	R	14	2.84	0.06	45, 29, −14
Effects of GLP-1 receptor blockade on meal effects							
Food > non-food	Healthy controls: meal reducing effects placebo > ex9-39	Insula	R	33	2.70	0.08	36, −13, 7
High-energy > non-food		–	–	–	–	–	–
Food > non-food	T2DM: meal reducing effects placebo > ex9-39	Insula	R	38	3.18	0.02	48, 8, 4
		Insula	L	22	3.10	0.03	−27, 26, 1
High-energy > non-food		OFC	R	11	3.00	0.04	48, 29, −11
		Caudate nucl.	L	15	2.79	0.06	−12, 23, 1
		Insula	L	16	2.69	0.08	−27, 26, 1

This table describes the areas where significant differences in CNS activations were observed for the three comparisons (group differences, effects of meal intake and effects of blockade of the GLP-1 receptor in both groups). For each comparison, the two contrasts (activation during food > non-food pictures and high-energy food > non-food pictures) are presented. The areas with significant differences are listed, including the cluster size of this effect, the Z value and the FWE corrected *p* value after small volume correction. The last column describes the coordinates of the peak voxel of the observed difference in MNI space

Caudate nucl. caudate nucleus; ex9-39, exendin 9-39; FWE, family-wise error; L, left; MNI, Montreal Neurological Institute; R, right; T2DM, obese type 2 diabetes patients

### Increased CNS activation in response to viewing food pictures in obese patients with type 2 diabetes vs healthy lean individuals

**Fig. 2:**
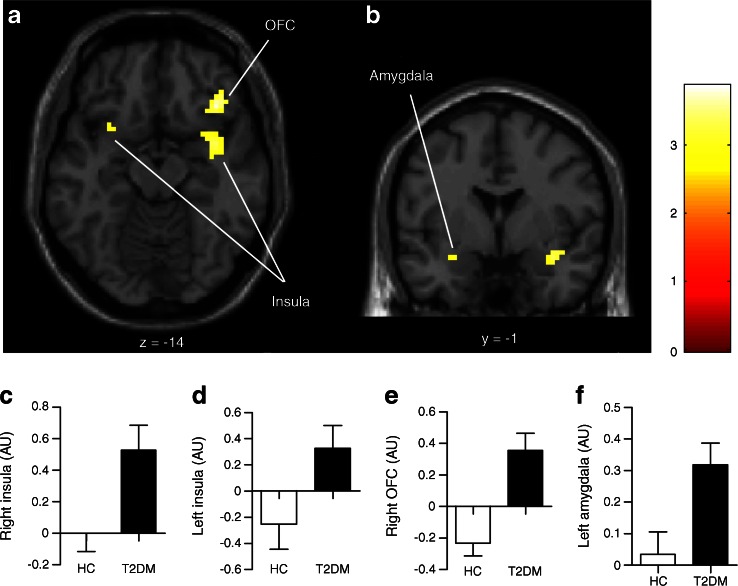
Between-group differences on CNS activation in response to viewing food pictures. (**a**) Axial and (**b**) coronal slices showing average differences in activation in brain regions where patients with diabetes vs healthy lean individuals had hyperactivation in response to viewing food pictures. The colour scale reflects the T-value of functional activity. Results are presented at the threshold of *p* < 0.05, FWE corrected on cluster extent. In the graphs, the BOLD signal intensity (effect size [AU]) for each group is plotted as mean and SEM for (**c**) the right and (**d**) left insula, (**e**) right OFC and (**f**) left amygdala. AU, arbitrary units; FWE, family-wise error; HC, healthy lean controls/individuals; T2DM, type 2 diabetes patients

During the session with placebo infusion in the fasted condition, obese patients with type 2 diabetes showed increased activation in the right OFC (*p* = 0.004), left amygdala (*p* = 0.02) and bilateral insula (right *p* = 0.02 and left *p* = 0.04, respectively) in response to food pictures (Fig. 2) and in the right OFC (*p* = 0.05) and left insula (*p* = 0.04) in response to high-energy food pictures. However, in the postprandial condition, increased activation in patients with diabetes was no longer observed in any brain area studied. In addition, we did not observe increased activation in healthy lean individuals compared with patients with diabetes in any of the ROIs either in the fasted or in the postprandial condition.

### Meal intake reduced CNS activation in response to food pictures

**Fig. 3:**
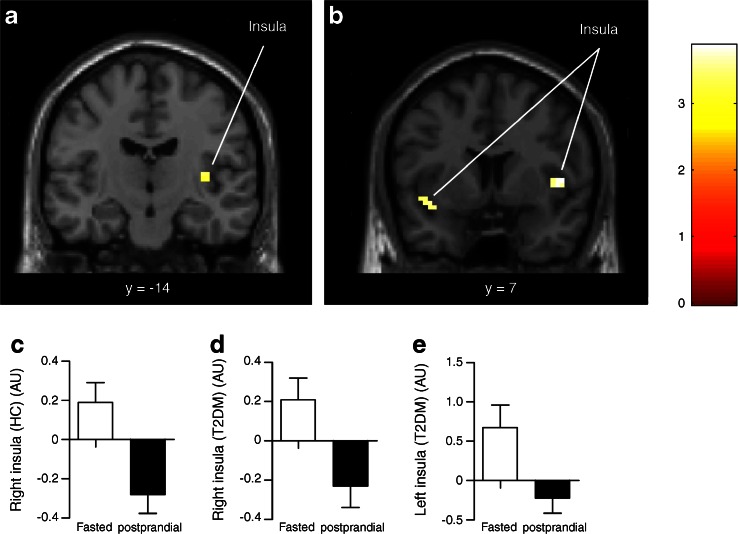
Meal intake effects on CNS activation in response to viewing food pictures. Coronal slices showing areas where intake of the meal reduced activation in response to viewing food pictures 30 min after intake in (**a**) healthy lean individuals and (**b**) obese patients with diabetes. The colour scale reflects the T-value of functional activity. Results are presented at the threshold of *p* < 0.05, FWE corrected on cluster extent. In the graphs, the BOLD signal intensity (effect size [AU]) mean and SEM is plotted for healthy lean individuals in (**c**) the right insula and for patients with diabetes in (**d**) the right and (**e**) left insula. AU, arbitrary units; FWE, family-wise error; HC, healthy lean controls/individuals; T2DM, type 2 diabetes patients

During placebo infusion, both groups showed reduced CNS activation in response to food pictures in the postprandial condition compared with the fasted condition. In healthy lean individuals, this effect of meal intake was observed in the right insula in response to food pictures (*p* = 0.02; Fig. 3a). In addition, meal intake tended to reduce activation in the right insula in response to the high-energy food pictures in lean healthy individuals (*p* = 0.08). In obese patients with type 2 diabetes, CNS activation was also reduced after meal intake in the bilateral insula in response to viewing food pictures (right *p* = 0.04, left *p* = 0.05; Fig. 3b) and in the left insula (*p* = 0.04), left caudate nucleus (*p* = 0.007) and right OFC (*p* = 0.06) in response to viewing high-energy food pictures. The effect of meal intake was more pronounced in patients with diabetes compared with healthy lean individuals in the right insula (*p* = 0.008) and bilateral OFC (right *p* = 0.01, left *p* = 0.03) in response to food pictures.

### Blockade of the GLP-1 receptor prevents effects of meal intake on CNS activation in response to food pictures

**Fig. 4:**
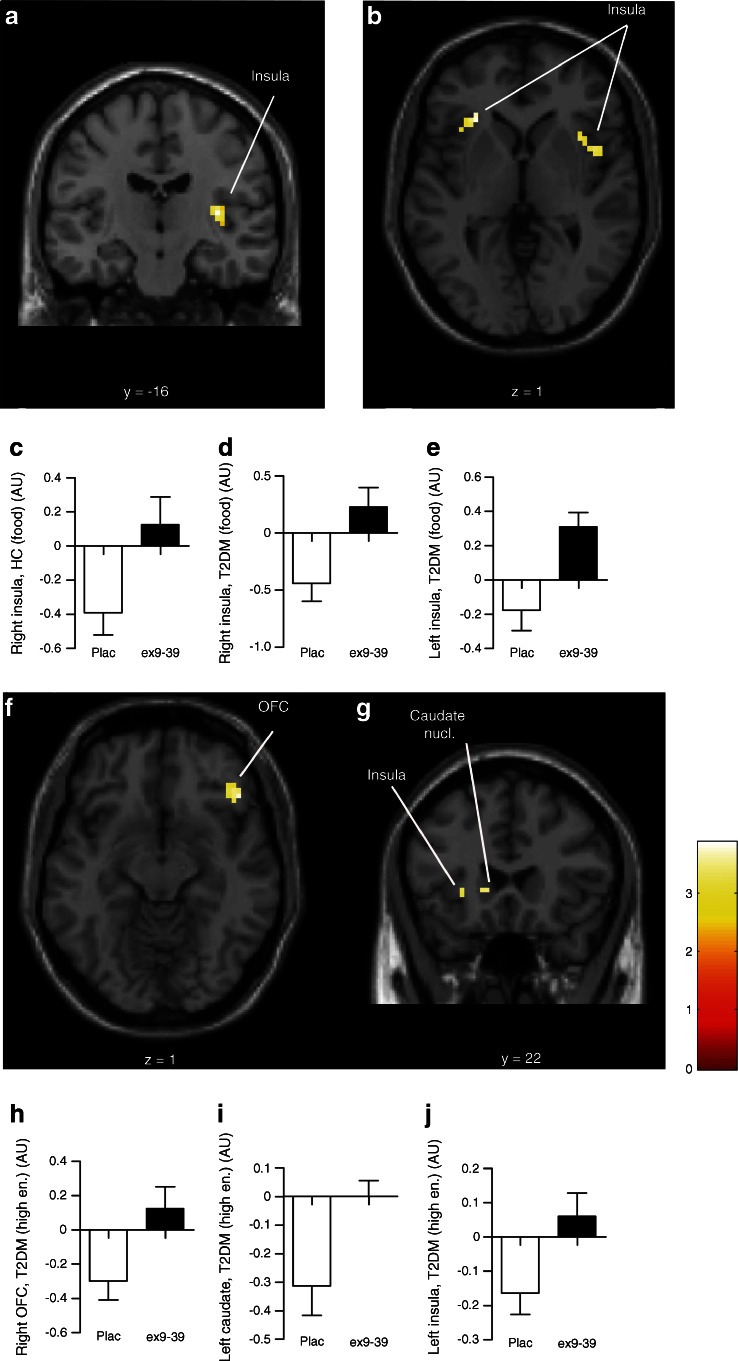
Effects of GLP-1 receptor blockade on CNS responses. Axial and coronal slices showing average differences in activation in brain regions where blockade of endogenous GLP-1 effects with exendin 9-39 prevented reducing effects of meal intake on activation to viewing food pictures in (**a**) healthy lean individuals (right insula *p* = 0.08) and (**b**) patients with type 2 diabetes (bilateral insula *p* < 0.05). The colour scale reflects the T-value of functional activity. In the graphs, the BOLD signal intensity (effect size [AU]) mean and SEM for healthy lean individuals in (**c**) the right insula and in patients with diabetes in (**d**) the right and (**e**) left insula. The effect of exendin 9-39 in patients with diabetes in response to viewing high-energy food pictures is shown for (**f**) the right OFC (*p* = 0.04) and (**g**) left caudate nucleus (*p* = 0.06) and left insula (*p* = 0.08). In the graphs, the signal intensity is plotted for (**h**) the right OFC, (**i**) left caudate nucleus and (**j**) left insula. AU, arbitrary units; ex9-39, exendin 9-39; high en., high-energy food pictures; plac, placebo

In healthy lean individuals, the effect of GLP-1 receptor blockade tended to be statistically significant in the right insula (*p* = 0.08), indicating that the reducing effect of meal intake on CNS activation in response to viewing food pictures may have been blunted by GLP-1 receptor blockade. In obese patients with type 2 diabetes, however, the reducing effect of meal intake on CNS activation was largely prevented in the bilateral insula by GLP-1 receptor blockade in response to viewing food pictures (right *p* = 0.04, left *p* = 0.03). In addition, the reducing effect of meal intake on CNS activation was prevented by the GLP-1 receptor blockade in the right OFC (*p* = 0.04) and tended to be prevented in the left caudate nucleus (*p* = 0.06) and left insula (*p* = 0.08) in response to viewing high-energy food pictures (Fig. 4).

### Appetite-related scores

In the healthy lean group, no significant differences in postprandial changes in any of the appetite-related scores were observed between the two experimental days (data not shown). However, in the patients with diabetes, exendin 9-39 infusion prevented the postprandial reductions in the scores for hunger that were observed with placebo 60 min after meal intake (mean ± SEM −2.3 ± 0.7 during placebo vs −1.1 ± 0.4 during exendin 9-39; *p* = 0.02). This effect was not significant at 30 min after meal intake (*p* = 0.5). Postprandial changes in the other scores did not differ significantly between the infusions.

### Blood glucose and plasma hormone levels

**Fig. 5:**
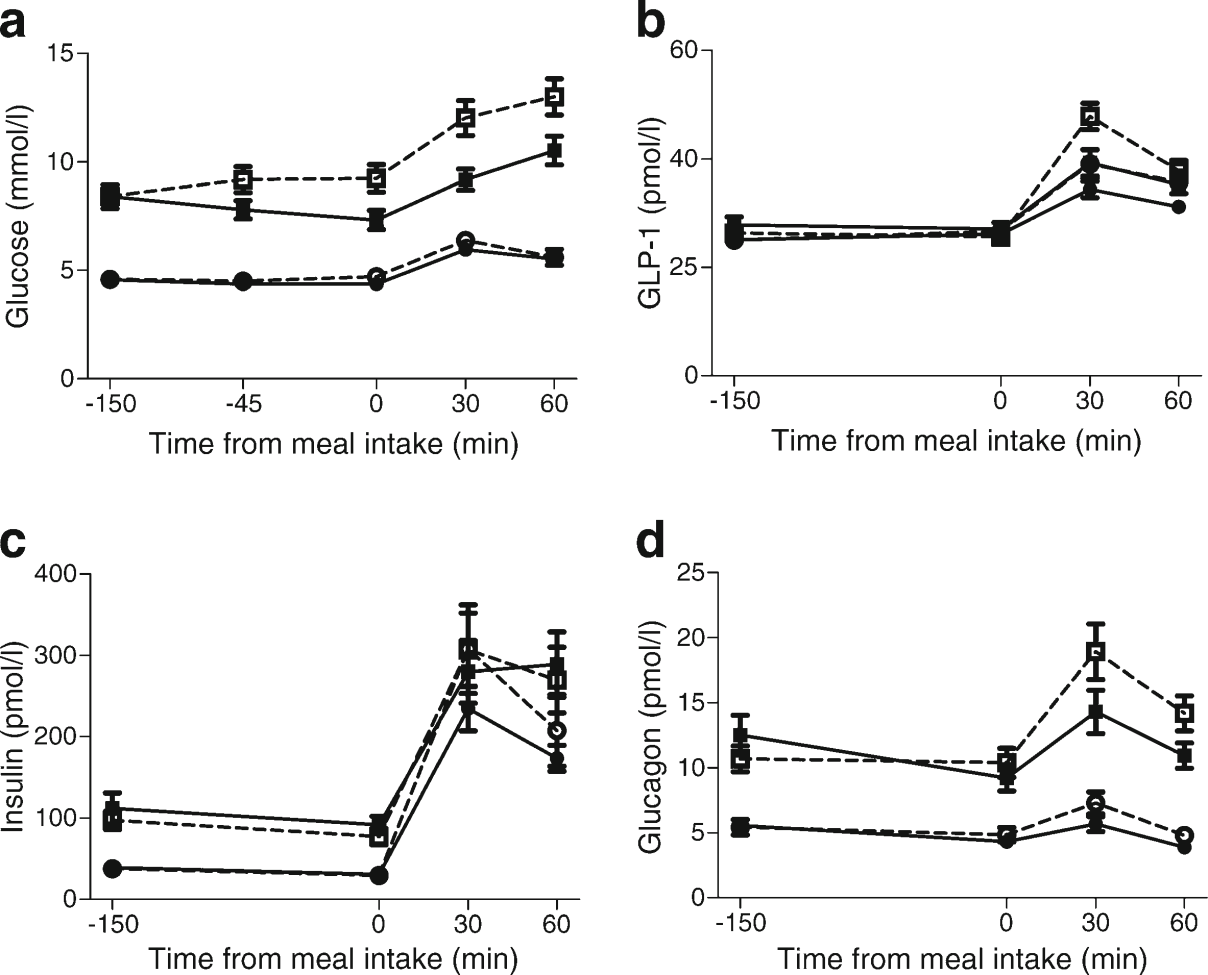
Glucose and plasma hormone levels. Levels of (**a**) glucose, (**b**) total GLP-1, (**c**) insulin and (**d**) glucagon during placebo (black) and exendin 9-39 (white) infusion in healthy lean individuals (circles) and obese patients with diabetes (squares). Data are mean ± SEM. Glucose levels were higher in diabetic patients vs healthy lean individuals (*p* < 0.001). Exendin 9-39 administration had no effect on glucose levels in healthy lean individuals (*p* = 0.4), but increased glucose levels in diabetic patients (*p* = 0.001). GLP-1 levels were higher during exendin 9-39 vs placebo administration (healthy, lean *p* = 0.04; diabetes *p* = 0.002). Insulin levels did not differ between groups nor between infusions in both groups (*p* ≥ 0.09). Glucagon levels were significantly higher in diabetic vs healthy lean individuals, and in both groups during exendin 9-39 vs placebo administration (*p* ≤ 0.004)

Figure 5 shows the glucose and hormone responses during both test visits. Glucose levels were significantly higher in patients with type 2 diabetes compared with healthy lean individuals during both placebo and exendin 9-39 administration (*p* < 0.001 for both). Compared with placebo, exendin 9-39 had no effect on glucose levels in healthy lean individuals (*p* = 0.4), whereas in patients with diabetes, glucose levels were significantly higher throughout the test visit with exendin 9-39 compared with placebo (*p* < 0.001). There were no significant differences in GLP-1 levels between healthy lean individuals and patients with diabetes during placebo infusion (*p* = 0.2), but in both groups, GLP-1 levels were significantly higher during exendin 9-39 infusion (*p* = 0.04 and *p* = 0.002 vs placebo in healthy lean individuals and patients with diabetes, respectively). Insulin levels did not differ between groups during placebo infusion (*p* = 0.3), and were unaffected by exendin 9-39 (*p* = 0.09 and *p* = 0.4 vs placebo in healthy lean individuals and patients with diabetes, respectively). During placebo infusion, glucagon levels were significantly higher in patients with diabetes compared with healthy lean individuals (*p* = 0.004), with levels being increased by exendin 9-39 compared with placebo (*p* = 0.004 and *p* < 0.001, respectively).

### Adverse events

Four individuals experienced abdominal discomfort 1–2 h after intake of the liquid meal (*n* = 2 during both visits, *n* = 1 during placebo and *n* = 1 during exendin 9-39 administration). One individual vomited shortly after the intake of the liquid meal on the visit with exendin 9-39. One individual experienced dizziness after the first fMRI session with exendin 9-39 for approximately 10 min.

## Discussion

Using fMRI we observed that while viewing food pictures, obese patients with type 2 diabetes display hyperactivation in CNS areas involved in the regulation of food intake. Furthermore, meal intake reduced CNS activation in healthy lean individuals and in obese patients with type 2 diabetes, but this effect was more pronounced in patients with diabetes. We found that in patients with diabetes, the GLP-1 receptor antagonist exendin 9-39 significantly prevented the reducing effect of meal intake on CNS activation. This finding provides the first evidence in humans for an effect of endogenous GLP-1 on CNS activation in areas involved in the regulation of feeding, supporting the concept that GLP-1 has a physiological role in the central regulation of feeding.

Animal and human studies have demonstrated that administration of pharmacological amounts of GLP-1RA results in reduced appetite, food intake [4–7] and body weight [8], and these effects are at least partly mediated by effects on the CNS [9–14, 19]. However, the physiological role of GLP-1 in the central regulation of feeding is less clearly established. Although not found consistently [25], treatment with the selective GLP-1 RA exendin 9-39 [17, 18, 26, 27] has resulted in significant increases in food intake [16]. In addition, in rats, central endogenous GLP-1 affects food intake and body weight [11, 18]. In humans, the effects of endogenous GLP-1 on prospective food consumption have been demonstrated [17], but changes in food intake could not be detected in a small pilot study [28]. Regarding the effects of endogenous GLP-1 on the CNS in humans, an association between postprandial increases in GLP-1 levels and the cerebral blood flow in areas involved in feeding behaviour has been observed [29], but our study is the first to investigate the effects of endogenous GLP-1 in an interventional setting.

In the current study, we showed that blockade of endogenous GLP-1 prevents the effects of meal intake on activation in the insula in response to the viewing of food pictures. The insula is known to be involved in the processing and evaluation of food cues and in craving for food [30, 31]. Blocking the actions of endogenous GLP-1 during the viewing of high-energy pictures also affected activation in the OFC and caudate nucleus, which are known to be involved in the process of reward evaluation [32]. In line with this finding, studies in rodents suggest that GLP-1 may decrease the rewarding effect of food by acting on central reward circuits [33, 34].

GLP-1 secreted from the intestine may access the brain through areas with a permeable blood–brain barrier. However, due to its short circulating half-life [35], it is likely that only a small amount of gut-derived endogenous GLP-1 reaches the brain. Therefore, it has been suggested that central effects of GLP-1 may also be mediated by indirect routes, such as vagal afferents originating from the intestine where GLP-1 levels are much higher [36]. In our study, we are not able to distinguish the direct from indirect effects.

The effects of endogenous GLP-1 on the CNS in our study might be explained by concomitant GLP-1 induced glucometabolic or hormonal changes. Glucose and glucagon have satiating effects that may be mediated by the CNS [37, 38]. However, despite the higher glucose and glucagon levels, we observed higher activation in the patients with diabetes compared with healthy lean individuals and higher CNS activation following exendin 9-39 administration compared with placebo. Hence, differences in glucose and glucagon levels cannot explain our findings and neither can insulin levels, which did not differ between the groups nor between infusions. Despite higher GLP-1 levels during exendin 9-39 administration, we observed that exendin 9-39 blocked GLP-1 effects.

In the healthy lean individuals, we were not able to detect a significant effect of postprandial endogenous GLP-1 on activation in CNS reward and satiety circuits. Healthy lean individuals showed lower CNS activation during the presentation of food pictures, possibly reducing the power to detect alterations due to endogenous GLP-1. Similarly, in a previous study, we were able to detect effects of pharmacological levels of GLP-1RA in healthy (normoglycaemic) obese individuals and obese patients with diabetes, whereas we [19] and others [39] were not able to detect these effects in healthy lean individuals. In accordance, the effect of meal intake on CNS activation in healthy lean individuals in the present study was much weaker than in patients with type 2 diabetes.

A limitation of this study is that we included only a group of healthy (normoglycaemic) lean individuals and obese patients with type 2 diabetes. We are, therefore, unable to distinguish the effects of obesity from diabetes per se. Although extrapolation of our findings in obese patients with diabetes to healthy (normoglycaemic) obese individuals awaits empirical confirmation, we believe that our findings may extend to healthy obese individuals for several reasons. First, in a previous study we showed that CNS activation in response to viewing food pictures was similarly increased in healthy obese individuals and in obese patients with type 2 diabetes [19]. Second, in this same study, we found that acute GLP-1RA administration reduced CNS activation in response to food pictures and reduced food intake in both healthy obese individuals and in obese patients with diabetes [19]. Third, several studies have shown that the effects of GLP-1RA treatment on body weight and food intake are similar in healthy obese individuals and in obese patients with diabetes [8, 40–43]. Fourth, in accordance with the findings in our study in obese patients with type 2 diabetes, others observed that GLP-1 administration at physiological levels in healthy obese men (without diabetes) resulted in decreased ratings of hunger and prospective food consumption [44]. Finally, in the present study, we demonstrated statistically significant effects of endogenous GLP-1 on CNS activation not only in obese patients with diabetes but also a trend in healthy lean individuals, suggesting that these effects are not confined to obese patients with diabetes.

Altered CNS activation after gastric distention has been described in obese patients [45], which may underlie their ability to consume large food volumes. However, in the current study, scanning was performed during rest only. Gastric distension may alter CNS baseline activity, but not necessarily activation differences when viewing food vs non-food pictures, as in the present study.

The reducing effect of GLP-1RA on gastric emptying is well-known [46, 47]. It could be speculated that a difference in the rate of gastric emptying between placebo and exendin 9-39 may have influenced our fMRI results. One study reported that exendin 9-39 has a small but significant effect on gastric emptying [48]. However, we performed the postprandial fMRI 30 min after meal intake, while the reported effect of exendin 9-39 on gastric emptying only started 45 min after intake [48]. In addition, others did not report altered gastric emptying by GLP-1 receptor blockade [49, 50].

In conclusion, our findings provide the first evidence that endogenous GLP-1 mediates satiating effects in areas of the CNS involved in satiety and reward in obese patients with type 2 diabetes. These data provide further insight into the central effects of peripheral signals, relaying information to the CNS and affecting feeding behaviour. Increased understanding of these processes may contribute to the development of new treatment strategies for obesity.

## Abbreviations

BOLD: Blood oxygen level dependent
CNS: Central nervous system
fMRI: Functional magnetic resonance imaging
GLP-1: Glucagon-like peptide-1
GLP-1RA: Glucagon-like peptide-1 receptor agonist
OFC: Orbitofrontal cortex
ROI: Region of interest
